# Developmental Exposure to the Flame Retardant Mixture Firemaster 550 Compromises Adult Bone Integrity in Male but not Female Rats

**DOI:** 10.3390/ijms21072553

**Published:** 2020-04-07

**Authors:** Soraia Macari, Kylie D. Rock, Mariana S. Santos, Virgínia T. M. Lima, Raphael E. Szawka, Jamal Moss, Brian Horman, Heather B. Patisaul

**Affiliations:** 1 Department of Restorative Dentistry, Federal University of Minas Gerais, Belo Horizonte, Minas Gerais 31270-901, Brazil; soraiamacari@gmail.com (S.M.); vilima15@hotmail.com (V.T.M.L.); 2 Department of Biological Sciences, North Carolina State University, Raleigh, NC 27695, USA; kdrock@ncsu.edu (K.D.R.); jemoss@ncsu.edu (J.M.); bmhorman@ncsu.edu (B.H.); 3 Department of Morphology, Federal University of Minas Gerais, Belo Horizonte, Minas Gerais 31270-901, Brazil; marianadesouzaz@gmail.com; 4 Department of Physiology and Biophysics, Federal University of Minas Gerais, Belo Horizonte, Minas Gerais 31270-901, Brazil; 5 Center for Human Health and the Environment, North Carolina State University, Raleigh, NC 27695, USA

**Keywords:** obesogen, endocrine disrupting chemicals, sex difference, adipose, trabecular, cortical bone, ppar gamma

## Abstract

Flame retardants (FRs) are used in a variety of common items from furniture to carpet to electronics to reduce flammability and combustion, but the potential toxicity of these compounds is raising health concerns globally. Organophosphate FRs (OPFRs) are becoming more prevalent as older, brominated FRs are phased out, but the toxicity of these compounds, and the FR mixtures that contain them, is poorly understood. Work in a variety of in vitro model systems has suggested that FRs may induce metabolic reprogramming such that bone density is compromised at the expense of increasing adiposity. To address this hypothesis, the present studies maternally exposed Wistar rat dams orally across gestation and lactation to 1000 µg daily of the FR mixture Firemaster 550 (FM 550) which contains a mixture of brominated FRs and OPFRs. At six months of age, the offspring of both sexes were examined for evidence of compromised bone composition. Bone density, composition, and marrow were all significantly affected, but only in males. The fact that the phenotype was observed months after exposure suggests that FM 550 altered some fundamental aspect of mesenchymal stem cell reprogramming. The severity of the phenotype and the human-relevance of the dose employed, affirm this is an adverse outcome meriting further exploration.

## 1. Introduction

Chemical flame retardants (FRs) are commonly applied to polymers, foam, resins, electronics and construction materials to reduce their flammability or delay their combustion. Because they are not chemically bound, they readily escape and accumulate in indoor dust. Over the past few decades, it has become increasingly apparent that some of these FRs have toxic properties. The FR mixture Firemaster^®^ 550 (FM 550) entered commercial use in the early 2000s and rapidly became one of the most commonly detected FRs in polyurethane foam used and sold in the US [1,2,3,4]. FM 550 contains a mixture of organophosphates: (1) triphenyl phosphate (TPHP; previously abbreviated TPP); and (2) a mixture of isopropylated triphenylphosphate isomers (ITPs); as well as two brominated compounds (3) 2-Ethylhexyl-2,3,4,5-tetrabromobenzoate (TBB); and (4) bis(2-Ethylhexyl)-2,3,4,5-tetrabromophthalate (TBPH). Work by us and others have identified this mixture to be developmentally neurotoxic, endocrine disrupting, and obesogenic [5,6,7,8,9,10,11,12,13]. Human exposure is nearly ubiquitous with both gestational and lactational transfer as significant exposure routes in addition to direct exposure via household dust, which is inadvertently ingested or inhaled [14,15,16,17]. Additionally, the individual components are present in other FR mixtures, and many other commonly used products. For example, TPHP is used in nail polish [18] and has rapidly become one of the most abundant and frequently detected organophosphate flame retardants (OPFRs) in indoor dust globally, including samples from the U.S., Egypt, Japan, Germany, and Portugal [3,19,20,21,22]. Thus, diphenyl phosphate (DPHP), the primary metabolite of TPHP, is nearly ubiquitous in human urine samples [23,24,25,26,27,28,29].

Prior work has shown that FM 550 components bind and activate peroxisome proliferator-activated receptor γ (PPARγ) [9,12] and promote mature adipocyte differentiation while diverting murine bone marrow multipotent mesenchymal stromal cell (MSC) differentiation away from osteogenesis [10]. These data suggest that FM 550 exposure could concomitantly result in obesity and loss of bone density. PPARγ is a master regulator of adipogenesis and a central player in metabolic syndrome [30]. A pilot study conducted in our lab with Wistar rats confirmed that perinatal exposure (oral to the dam) to 1000 µg FM 550 significantly enhanced adult body weight, particularly in males [6]. In the heart, left ventricle thickness was also significantly increased in exposed males, suggesting impaired cardiovascular performance. The present study used bone tissue from animals derived from a subsequent, higher powered, study focused on neuroendocrine endpoints [8] to test for effects on bone density and microarchitecture with the hypothesis that males would be more vulnerable than females.

## 2. Results

At the time of euthanasia, body weight of the exposed females (323.3 ± 7.9 g) was significantly higher than control females (292.2 ± 4.8 g; *t* = 3.56; *p* < 0.01). In males, body weight in the controls (620.0 ± 33.1) did not significantly differ from the exposed group (578.4 ± 11.3; *t* = 1.28; *p* = 0.2). 

**Figure 1:**
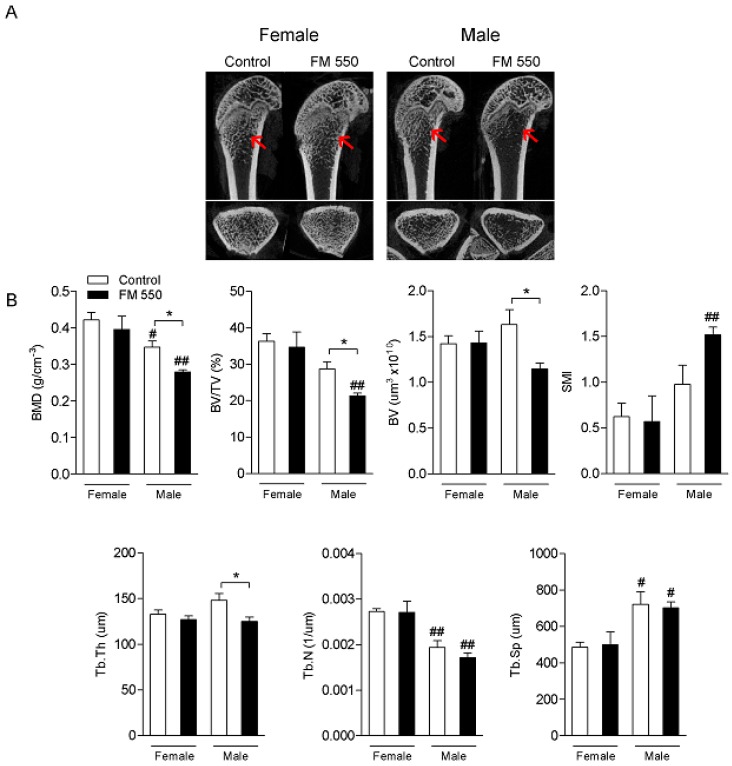
The flame retardant (FR) mixture Firemaster 550 (FM 550) induces long-term bone loss in the femur trabecular bone of male but not female rats as demonstrated by decreased bone microarchitecture. (**A**) Representative micro computed-tomography (microCT) images of female (left) and male (right) rats from Control and exposed to FM 550 groups. Longitudinal (top) and transversal (bottom) views of the femur are shown. Red arrows indicate the metaphysis region in which bone parameters were analyzed; (**B**) Mean ± standard error of the mean (SEM) of bone mineral density (BMD; g/cm^−3^), percent bone volume/tissue volume (BV/TV; %), bone volume (BV; µm^3^), structural model index (SMI), trabecular thickness (Tb.Th; µm), trabecular number (Tb.N; 1/µm), and trabecular separation (Tb.Sp; µm). Two-way ANOVA followed by the Holm–Sidak post hoc test (*n* = 4 per group). * *p* < 0.05 different from control of same sex; # *p* < 0.05 different from exposed females, ## *p* < 0.01 different from exposed females.

For every bone structure endpoint assessed, only males were significantly affected. MicroCT analysis of femoral epiphysis is shown in Figure 1. As determined by two-way ANOVA, males displayed lower BMD (F1,12 = 19.87; *p* < 0.001), BV/TV (F1,12 = 19.60; *p* < 0.001), and Tb.N (F1,12 = 32.62; *p* < 0.001) alongside greater SMI (F1,12 = 11.18; *p* < 0.01) and Tb.Sp (F1,12 = 16.64; *p* < 0.01) compared with females. Femoral BMD (F1,12 = 5.85; *p* < 0.05), BV/TV (F1,12 = 5.00; *p* < 0.05), BV (F1,12 = 4.50; *p* = 0.055), and Tb.Th (F1,12 = 6.87; *p* < 0.05) were significantly decreased in FM 550-exposed males compared to unexposed controls. The SMI value showed a trend toward an increase in FM 550-exposed males compared with the respective controls, but the effect did not reach statistical significance (*p* = 0.07). In spite of being sexually dimorphic, Tb.N (*p* = 0.44) and Tb.Sp (*p* = 0.96) were not affected by FM 550 exposure.

**Figure 2:**
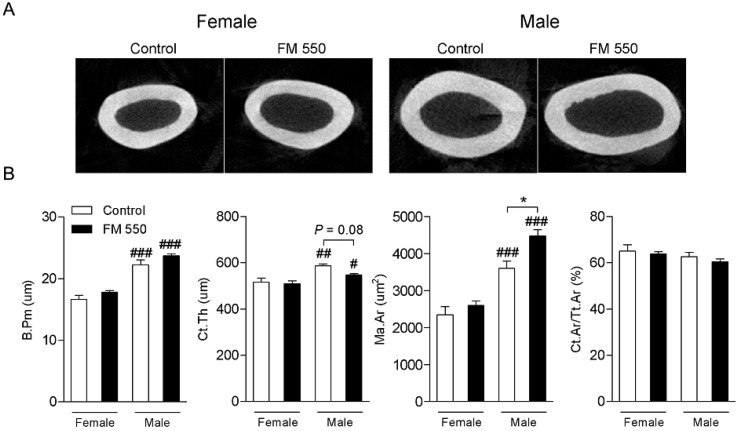
Developmental effect of FM 550 on the femur cortical bone of female and male rats. (**A**) Representative microCT images of cross-sectional cortical bone female (left) and male (right) rats from Control and exposed to FM 550 groups. (**B**) Mean ± SEM of bone perimeter (B.Pm; µm), cortical thickness (Ct.Th; µm), cortical bone area/total tissue area (Ct.Ar/Tt.Ar; %), medullary area (Ma.Ar; µm^2^). Two-way ANOVA followed by the Holm-Sidak post hoc test (*n* = 4 per group). * *p* < 0.05 different from control of same sex, # *p* < 0.05 different from exposed females, ## *p* < 0.01 different from exposed females, ### *p* < 0.001 different from exposed females.

MicroCT analysis of femoral cortical bone is shown in Figure 2. As determined by two-way ANOVA, B.Pm (F1,12 = 111.8; *p* < 0.001), Ct.Th (F1,12 = 20.66; *p* < 0.001), and Ma.Ar (F1,14 = 75.23; *p* < 0.001) were all greater in males than in females, which was anticipated given that males are physically larger than females. Ma.Ar was increased in FM 550-exposed males compared with controls (F1,14 = 9.77; *p* < 0.01). Moreover, although not reaching statistical significance, there was a trend toward reduction in the Ct.Th of male rats exposed to FM 550 (F1,12 = 3.58; *p* = 0.08).

**Figure 3:**
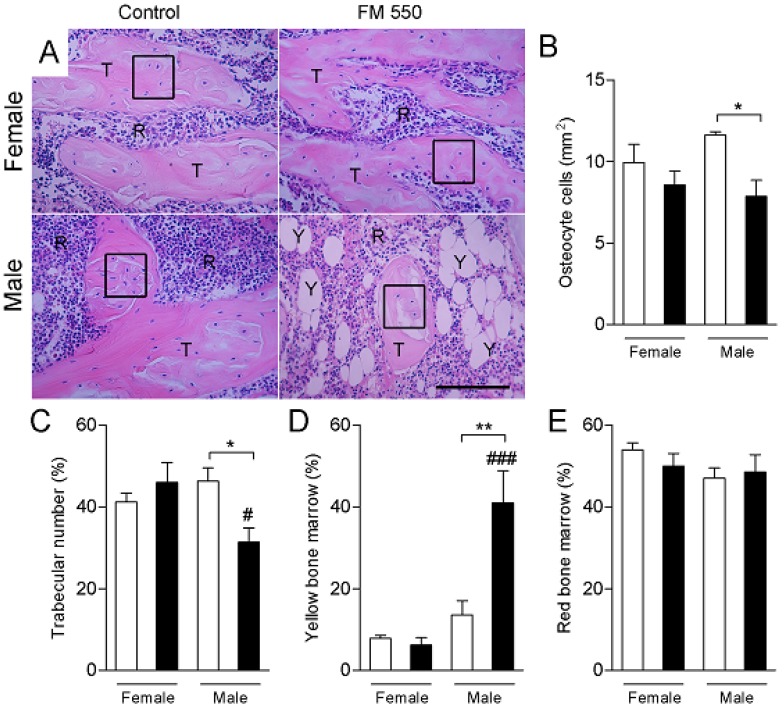
Developmental exposure to FM 550 attenuates bone cell differentiation in favor of adipocytes in male rats. Histomorphometric analysis of the femurs was performed using hematoxylin and eosin. (**A**) Representative images of hematoxylin and eosin staining in female (top) and male (bottom) rats exposed during development to FM 550. Black oblongs indicate the area of osteocytes count. T, trabecular bone. R, red bone marrow. Y, yellow bone marrow. Scale bar, 100 µm. Mean ± SEM number of osteocyte cell (**B**), percentage of trabecular bone (**C**), percentage of yellow bone marrow (**D**), and percentage of red bone marrow (**E**). Two-way ANOVA followed by the Holm–Sidak post hoc test. (*n* = 4 per group). * *p* < 0.05 different from control of the same sex, ** *p* < 0.01 different from control of the same sex, # *p* < 0.05 different from exposed females, ### *p* < 0.001 different from exposed females.

**Figure 4:**
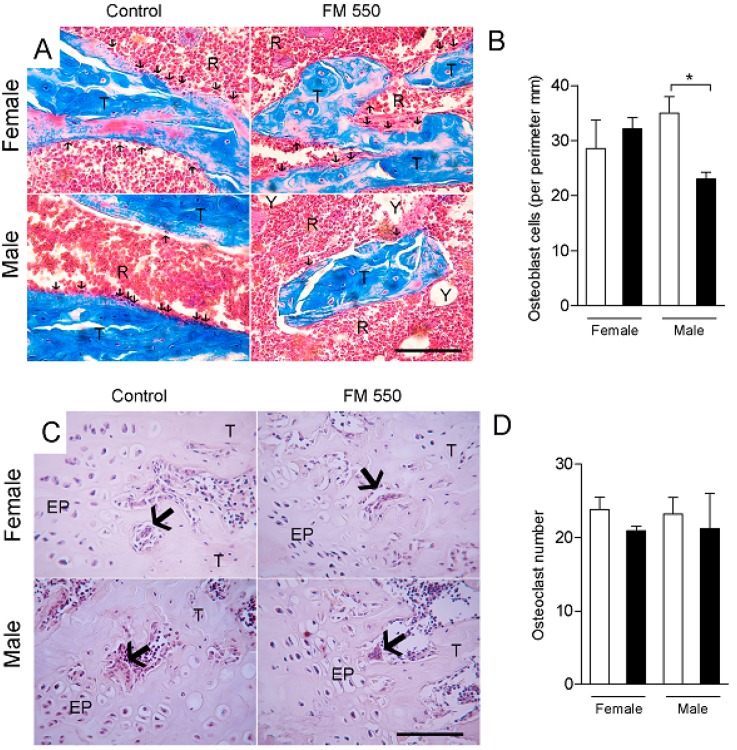
Developmental exposure to FM 550 decreases osteoblast number in male but not female rats. Histomorphometric analysis of the femurs using Masson’s trichrome or tartrate resistant acid phosphatase (TRAP) staining. (**A**) Representative images of Masson’s trichrome staining in female (top) and male (bottom) rats exposed during development to FM 550. Black arrows indicate osteoblasts cells along the trabecular bone. T, trabecular bone. R, red bone marrow. Y, yellow bone marrow. Scale bar, 100 µm; (**B**) Mean ± SEM number of osteoblast. Two-way ANOVA followed by the Holm–Sidak post hoc test (*n* = 4 per group). * *p* < 0.05 different from control of the same sex; (**C**) Representative images of TRAP-positive osteoclasts in female (top) and male (bottom) rats from Control and exposed to FM 550 groups. Large arrows indicate osteoclasts. EP, epiphyseal disc. T, trabecular bone. Scale bar, 100 µm; (**D**) Mean ± SEM number of TRAP-positive osteoclasts. Two-way ANOVA followed by the Holm–Sidak post hoc test (*n* = 4 per group) determined no effect of sex (*p* = 0.95) or FM 550 (*p* = 0.29).

Histomorphometric analyses of the femurs are shown in Figure 3 and Figure 4. In the hematoxylin and eosin staining, the number osteocytes (F1,12 = 8.72; *p* < 0.05) and percentage of trabecular bone (F1,12 = 7.75; *p* < 0.05) were both significantly decreased in FM 550-exposed males. FM 550 also markedly increased the percentage of yellow bone marrow in male rats (F1,12 = 8.49; *p* < 0.05; Figure 3). Masson’s trichrome staining revealed that FM 550 decreased the number osteoblasts in the femur (F1,11 = 5.26; *p* < 0.05; Figure 4A,B), whereas the number of TRAP-positive osteoclasts was not found to be affected by sex (F1,10 = 0.003; *p* = 0.95) or FM 550 (F1,10 = 1.25; *p* = 0.29; Figure 4C,D).

## 3. Discussion

Our present findings reveal, for the first time in vivo, that developmental exposure to the flame retardant mixture FM 550 can compromise bone health in adult male rats long after exposure ceases. This phenomenon, in combination with significantly elevated body weight and left ventricle thickness observed in a prior, similar, study [6], suggests that overall metabolic health may be impaired. These data are consistent with numerous in vitro studies suggesting that FM 550, its components, and structurally related FRs, heighten adiposity at the expense of bone formation [10,31,32,33,34,35,36,37,38,39,40,41,42,43].

Bone marrow mesenchymal stem/stromal cells (BMSCs) have multipotential to differentiate into osteoblasts, adipocytes, chondrocytes or myoblasts. Molecular factors and differing signaling pathways controlling transcription may alternatively trigger events that direct BMSCs to osteogenesis or adipogenesis [34]. In cases where BMSCs are not directed towards osteogenesis, bone formation may be impaired resulting in deficient bone microarchitecture. In our results, bone formation was deficient in FM550-exposed males, as demonstrated by the microCT analysis. This supports the hypothesis that early exposure to FM 550 attenuated bone differentiation in favor of adipocyte differentiation, which is suggested by the decrease in trabecular bone and the number of osteoblasts in opposition to the increase in yellow bone marrow percentage.

Because it is a mixture, and the components are rapidly metabolized to several different metabolites [2], it is not possible from this single study to ascertain which of the parent compounds or metabolites may have contributed to the observed effects. Data from a range of experimental systems, however, provide compelling evidence that the organophosphate components of FM 550 are likely the primary drivers of this phenotype. Experiments in murine 3T3-L1 preadipocytes revealed that FM550 and its components TPHP, IPTP, and TBPH, but not TBB, dose-dependently induces lipid accumulation as well as PPARγ-mediated adipocyte protein 2 (aP2) enhancer activity [35]. This upregulation was blocked by the PPARγ-selective antagonist GW9662. Chromatin immunoprecipitation further confirmed a role for PPARγ by revealing that IPTP and TPP induced recruitment of PPARγ to the regulatory region of aP2. Work in ex vivo fetal murine limb bud cultures also identified TPHP as particularly detrimental to bone formation via suppression of Sox9, Runx2 and Sp7; genes known to be regulated by PPARγ [31]. Similarly, FM 550, and its organophosphate components, induce adipogenesis in human primary preadipocytes via a mechanism that highly implicates PPARγ [35]. A recent study by the National Toxicology Program (NTP), identified TPHP as a “high priority” chemical meriting additional testing due to evidence of developmental and neurotoxic properties comparable to the brominated flame retardants (largely polybrominated diphenyl ethers or PBDEs) it was meant to replace [36]. While bone density and composition were not mentioned specifically, it cautions that modern FRs are structurally similar to, and likely just as toxic, as the chemicals they replaced. 

Notably, the present study is also consistent with prior work linking organophosphate pesticides with disrupted bone formation and maintenance [37]. Organophosphate pesticide studies may yield additional insight as to possible mechanisms of organophosphate FR action. For example, chlorpyrifos in combination with retinoic acid (RA), directs commitment of mesenchymal stem cells to adipogenic differentiation through a process involving a crosstalk at GSK3β signaling. Similarly, simultaneous subchronic oral exposure to selenium (5 mg) and diazinon (40 mg) in drinking water for 90 days induced changes in macroscopic and microscopic structures of adult male rat femurs including decreased femoral length and cortical bone thickness [38]. Because they contain acetylcholinesterase, MSC regulation and differentiation is thought to be disrupted by organophosphate pesticides via interference with acetylcholine signaling [39]. A role for sex has not been considered in much of the pesticide work, but should be a factor in future studies with FRs.

Importantly, an adverse role for the brominated compounds in the FM 550 FR mixture cannot be ruled out. Notably, two EPA-mandated studies conducted by Chemtura using only the brominated components of FM 550 (a two generation reproductive study (MPI Research Study 1038–008; “CN-2065: An Oral Two-Generation Reproduction and Fertility Study in Rats”) and a developmental toxicity study (MPI Research Study 1038–006; CN-2065:Prenatal Developmental Toxicity Study in Rats”) also found bone-related phenotypes, albeit at much higher doses. Exposures in both studies ranged from 15 to 300 mg/kg/day via oral gavage, which is typical for a guideline study conducted for regulatory purposes. Observations included fused cervical vertebral neural arches in fetuses of exposed dams, an effect attributed to exposure at the 300 mg/kg dose but classified as “incidental” at the 100 mg/kg dose. In both studies, the no observable adverse effects level (NOAEL) was set at 50 mg/kg/day, a level far exceeding typical human exposures and the dose used in the present study.

## 4. Materials and Methods

All tissues were obtained from animals used for a prior study, the detailed methods of which are published elsewhere [8] using the ARRIVE guidelines checklist (http://www.nc3rs.org.uk/ARRIVE). That study used three orally administered doses of FM 550. Femoral samples from the highest dose group (1000 µg per day; ~3.3 mg/kg bw to the dam) and sex-matched vehicle (ethanol) controls were used for the present study. Accuracy of FM 550 dosing solution was confirmed by gas chromatography mass spectrometry. All dosing and testing was done blinded to exposure group. 

Animal care and maintenance were conducted in accordance with the applicable portions of the Animal Welfare Act and the U.S. Department of Health and Human Services’ “Guide for the Care and Use of Laboratory Animals” and approved by the North Carolina State University (NCSU) Institutional Animal Care and Use Committee (protocol ID# 17-058-B under Animal Welfare Assurance #D16-00214). All animals were obtained from an existing colony of Wistar rats maintained in a reversed light environment that minimizes exogenous exposure to endocrine disrupting compounds (EDCs) at the NCSU Biological Resource Facility. Study animals were kept in a climate controlled room at 25 °C and 45–60% average relative humidity on a phytoestrogen free diet (Teklad 2020, Harlan), and housed in thoroughly washed polysulfone cages with glass water bottles (rubber stoppers and metal sippers) and woodchip bedding to minimize unintentional exposure to endocrine disrupting compounds [40,41]. 

Daily oral exposure of the dams (*n* = 8 in the control group and *n* = 7 in the 1000 µg FM 550 group because one dam failed to litter) spanned gestational day (GD) 8 through weaning (postnatal day (PND) 21) by dispensing 20 µL of ethanol-based solution containing the appropriate dose onto ¼ of a soy-free food treat pellet (apple or chocolate flavored AIN-76A Rodent Diet Test Tabs, Test Diet, Richmond, IN, USA). Oral dosing via food treats models human exposure while minimizing handling stress [42,43,44]. Litters were standardized to 10 (5:5 sex ratio whenever possible) on PND 1. On PND 21 pups were weaned into same sex littermate groups of up to 3 and housed in the same conditions as the dams. Animals were euthanized as adults (6 months of age), weighed, and the tissues distributed and archived for a variety of studies (detailed in Baldwin et al. [8]). Hind legs were surgically removed from a subset of animals at the time of euthanasia, frozen on dry ice and stored at −80 °C. Whole legs (*n* = 4 per sex per exposure group; one per sex per litter) were shipped to the Macari lab on dry ice for bone analyses.

### 4.1. Micro Computed-Tomography (microCT) Imaging

The femur was analyzed by microCT as previously described [45]. Briefly, femurs were dissected and fixed in 10% buffered formalin (pH 7.4) and samples were scanned using a compact desktop microCT scanner Skyscan 1174 (Bruker-MicroCT, Kontich, Belgium), with 50 kV of source voltage, 800 µA source current and 12.18 µm pixel size. A 0.5 mm aluminum filter was used. The images were reconstructed by NRecon software (Bruker-MicroCT, Kontich, Belgium), the position of the sample was determined by Dataviewer software (Bruker-MicroCT, Kontich, Belgium) and analyzed using CTan software (Bruker-MicroCT, Kontich, Belgium). An irregular and anatomic shaped region of interest was used as a contouring method to delineate the region of interest adjacent to the endocortical boundary in the metaphyseal region of distal femurs [46]. The trabecular bone was analyzed to determine bone mineral density (BMD g/cm^−3^), percent bone volume/tissue volume (BV/TV %), bone volume (BV µm^3^), structural model index (SMI), trabecular thickness (Tb.Th µm), trabecular number (Tb.N 1/µm) and trabecular separation (Tb.Sp µm). The cortical bone was examined for bone perimeter (B.Pm µm), cortical bone area/total tissue area (Ct.Ar/Tt.Ar %), cortical thickness (Ct.Th µm) and medullary area (Ma.Ar um^2^).

### 4.2. Bone Histomorphometry

Measurements were performed at the distal femur excluding the growth plate as previously described [45]. The femurs were fixed in 10% formalin, decalcified in 14% EDTA and embedded in paraffin. Sagittal sections of 5 µm were stained using hematoxylin and eosin for density of osteocyte counting per trabecular bone area, tartrate resistant acid phosphatase (TRAP; Sigma-Aldrich, Saint Louis, MO, USA) for osteoclast TRAP positive cells analysis, and Masson’s trichrome staining for osteoblast counting per trabecular bone perimeter. The percentages of red bone marrow, yellow bone marrow and trabecular number were determined at 40× magnification using an ocular micrometer containing a 121-point grid. Five sections per animal were evaluated under microscope (Olympus AX70 Light Microscope, Olympus Corporation, Tokyo, Japan).

### 4.3. Statistical Analysis

The litter was the statistical unit (one animal per sex per litter was used). Data are presented as the mean ± standard error of the mean (SEM). Body weight data were compared within sex by Student *t*-test. For bone data, differences among groups were analyzed by two-way ANOVA followed by the Holm–Sidak post hoc test. In all cases, *p* ≤ 0.05 was considered statistically significant.

## 5. Conclusions

Here we show, for the first time in vivo, that developmental exposure to FM 550, which contains TPHP and other suspected toxicants, adversely impacts bone health in adult males. The fact that the phenotype was observed months after exposure suggests that FM 550 altered some fundamental aspect of mesenchymal stem cell reprogramming, but this requires confirmation. While a small study with limited numbers, given the severity of the phenotype, use of different methodologies for bone analysis, consistency with prior in vitro data in multiple species, and the human-relevance of the dose employed, these data merit follow-up work to further explore this potential adverse outcome. Mode of action and possible methods of ameliorating this effect are of primary interest.
